# Lysosomal glycogen accumulation in Pompe disease results in disturbed cytoplasmic glycogen metabolism

**DOI:** 10.1002/jimd.12560

**Published:** 2022-10-17

**Authors:** Rodrigo Canibano‐Fraile, Laurike Harlaar, Carlos A. dos Santos, Marianne Hoogeveen‐Westerveld, Jeroen A. A. Demmers, Tim Snijders, Philip Lijnzaad, Robert M. Verdijk, Nadine A. M. E. van der Beek, Pieter A. van Doorn, Ans T. van der Ploeg, Esther Brusse, W. W. M. Pim Pijnappel, Gerben J. Schaaf

**Affiliations:** ^1^ Department of Clinical Genetics Erasmus MC University Medical Center Rotterdam The Netherlands; ^2^ Department of Pediatrics Erasmus MC University Medical Center Rotterdam The Netherlands; ^3^ Center for Lysosomal and Metabolic Diseases Erasmus MC University Medical Center Rotterdam The Netherlands; ^4^ Department of Neurology Erasmus MC University Medical Center Rotterdam The Netherlands; ^5^ Erasmus Center for Biomics Erasmus MC University Medical Center Rotterdam The Netherlands; ^6^ Department of Human Biology NUTRIM School of Nutrition and Translational Research in Metabolism, Maastricht University Medical Center Maastricht The Netherlands; ^7^ Princess Máxima Center for Pediatric Oncology Utrecht The Netherlands; ^8^ Department of Pathology, Section Neuropathology Erasmus MC University Medical Center Rotterdam The Netherlands

**Keywords:** glycogen metabolism, lysosomal storage disorder, metabolic myopathy, Pompe disease, skeletal muscle

## Abstract

Pompe disease is an inherited metabolic myopathy caused by deficiency of acid alpha‐glucosidase (GAA), resulting in lysosomal glycogen accumulation. Residual GAA enzyme activity affects disease onset and severity, although other factors, including dysregulation of cytoplasmic glycogen metabolism, are suspected to modulate the disease course. In this study, performed in mice and patient biopsies, we found elevated protein levels of enzymes involved in glucose uptake and cytoplasmic glycogen synthesis in skeletal muscle from mice with Pompe disease, including glycogenin (GYG1), glycogen synthase (GYS1), glucose transporter 4 (GLUT4), glycogen branching enzyme 1 (GBE1), and UDP‐glucose pyrophosphorylase (UGP2). Expression levels were elevated before the loss of muscle mass and function. For first time, quantitative mass spectrometry in skeletal muscle biopsies from five adult patients with Pompe disease showed increased expression of GBE1 protein relative to healthy controls at the group level. Paired analysis of individual patients who responded well to treatment with enzyme replacement therapy (ERT) showed reduction of GYS1, GYG1, and GBE1 in all patients after start of ERT compared to baseline. These results indicate that metabolic changes precede muscle wasting in Pompe disease, and imply a positive feedforward loop in Pompe disease, in which lysosomal glycogen accumulation promotes cytoplasmic glycogen synthesis and glucose uptake, resulting in aggravation of the disease phenotype.

## Graphical abstract

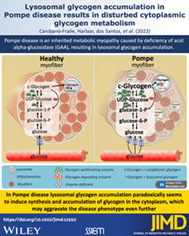

## 1 INTRODUCTION

Pompe disease (OMIM: no. 232300) is a rare metabolic myopathy characterized by acid alpha‐glucosidase deficiency caused by disease‐associated variants in the acid alpha‐glucosidase (GAA; EC 3.2.1.20) gene. As a consequence, glycogen cannot be degraded and accumulates in the lysosomes.^1^, ^2^, ^3^ In the most severe classic infantile form of Pompe disease, GAA enzyme activity is virtually absent and symptoms manifest shortly after birth, consisting of generalized skeletal muscle weakness and a hypertrophic cardiomyopathy.^2^ In patients with symptom onset at childhood or adulthood (late‐onset patients), a more slowly progressive skeletal muscle weakness develops resulting in impaired motor and respiratory function that can lead to wheelchair and ventilator dependency at any age.^2^, ^4^ Since 2006 enzyme replacement therapy (ERT) using alglucosidase alfa (Lumizyme/Myozyme®, Sanofi Genzyme) is available for Pompe disease. ERT improves survival of classic infantile patients and largely normalizes hypertrophic cardiomyopathy^5^, ^6^, ^7^ and improves muscle strength and stabilize respiratory function in patients with onset at childhood or adulthood, albeit with considerable interindividual variability in treatment response.^8^, ^9^

Glycogen biosynthesis and metabolism is largely dependent on (1) glucose entry into the cell, and (2) the action of glycogen biosynthetic and degradative enzymes. While most glucose is stored in the cytoplasm, part of it is located inside lysosomes. Degradation of glycogen in the cytoplasm takes place via degradative enzymes, while in the lysosomes, glycogen is hydrolyzed by GAA under acidic conditions. As such, glycogen metabolism is the result of a delicate balance between biosynthesis and degradation, a process that is disturbed in glycogen storage disorders (GSDs).

Previous studies found a number of enzymes involved in cytoplasmic glycogen metabolism that are dysregulated in a knockout mouse model of Pompe disease.^10^, ^11^ These studies showed that several components of glycogen biosynthesis—including glucose transporter 4 (GLUT4; SLC2A4; HGNC:11009), hexokinase (HK1; EC 2.7.1.1), UDP‐glucose, glycogenin (GYG1; HGNC:4699), and glycogen synthase (GYS1; EC 2.4.1.11) were upregulated, while activity of glycogen metabolizing enzymes like phosphorylase (PYGM; EC 2.4.1.1) were reduced in Pompe disease compared with wild‐type (WT) animals.^11^, ^12^ Surprisingly, the effect of this dysregulation suggested increased cytoplasmic glycogen levels in Pompe disease, increasing the availability of substrate for lysosomal glycogen and generating a predicted positive feedforward loop for cellular glycogen accumulation.^10^, ^11^ Treatment of mice with ERT resulted in reversal of the levels of GYS1 and GYG1.^11^ A multicenter study found extralysosomal glycogen accumulation in muscle biopsies from late‐onset patients.^13^ Notably, extralysosomal glycogen accumulation was not cleared by ERT,^13^ consistent with the optimal enzymatic activity of recombinant human GAA used in ERT to degrade glycogen at an acidic pH that is found in lysosomes but not in the cytoplasm.

These findings provide important insight into the pathobiology of Pompe disease and may have clinical implications. However, as the genetic background is known to affect pathophysiological parameters in mouse models of Pompe disease,^14^ it remains unclear if these observations are restricted to the mouse model used—most previous studies were performed using germline GAA knockout (*Gaa* ^−/−^) mice in a mixed (Bl6/129) background^11^, ^15^—as well as to the tissues and enzymes reported. Furthermore, it is unknown if glycogen metabolism is indeed dysregulated in human patients. In this study, we not only confirmed dysregulated glycogen metabolism in a mouse model of Pompe disease on a different genetic background,^16^ but extended the observations to additional tissues that are affected in Pompe disease—diaphragm (DP) and brain (BR). Glycogen metabolism was most strongly dysregulated in skeletal muscle—quadriceps femoris (QF) and DP—although heart (HRT) and BR were also affected. In addition, our study revealed upregulation of two additional glycogen metabolizing enzymes—glycogen branching enzyme 1 (GBE1; EC 2.4.1.18) and UDP‐glucose pyrophosphorylase (UGP2; EC 2.7.7.9). Analysis of tissues from animals at different ages demonstrated that glycogen metabolism was already dysregulated before the onset of muscle wasting, which in *Gaa* ^*−/−*^ in a FVB/N background strain of laboratory inbred mice, takes place between 15 and 25 weeks of age,^17^ suggesting that metabolic changes may contribute to disease progression. Paired analysis of skeletal muscle biopsies from five mildly affected Pompe patients before and after ERT showed—despite large variability between patients in expression of enzymes involved in glycogen metabolism—decreased expression of GYG1, GYS1, GBE1, and UPG2 in biopsies after ERT, suggesting that also in Pompe disease patients cytoplasmic glycogen metabolism may be disturbed, and that ERT could reverse this dysregulation.

## 2 RESULTS

### 2.1 Cytoplasmic glycogen metabolism in *Gaa* ^−/−^ mice

**FIGURE 1:**
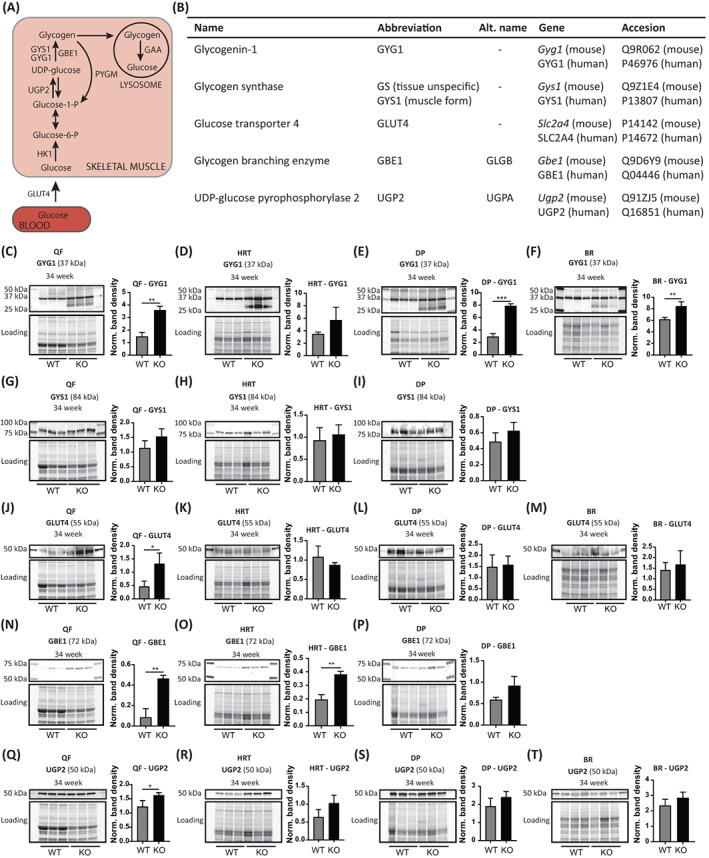
Expression of glycogen metabolizing enzymes in skeletal muscles, heart, and brain of adult *Gaa* ^−/−^ mice. (A) Diagram representing metabolic steps and enzymes involved in glycogen biosynthesis in skeletal muscle. (B) Summary of the nomenclature and accession numbers of the enzymes used in this study. (C–F) Western blot analyses and quantification of GYG1 in WT and *Gaa* ^−/−^ mice at 34 weeks in quadriceps femoris (QF), heart (HRT), diaphragm (DP), and brain (BR) lysates respectively. To quantify GYG1 levels, all bands between 50 and 30 kDa were used, as GYG1 is known to have a heterogeneous molecular weight due to its association to glycogen.^11^ Values from three independent mice were normalized to total protein and averaged. Data are shown as mean ± SE (*n* = 3) **p ≤ 0.01; ***p ≤ 0.001. (G–J) Western blot analyses and quantification of GYS1 in WT and *Gaa* ^−/−^ mice at 34 weeks in QF, HRT, and DP lysates respectively. Values from three independent mice were normalized to total protein and averaged. Data are shown as mean ± SE (*n* = 3). (K–M) Western blot analyses and quantification of GLUT4 in wild‐type (WT) and *Gaa* ^−/−^ mice at 34 weeks in QF, HRT, DP, and BR lysates respectively. Values from three independent mice were normalized to total protein and averaged. Data are shown as mean ± SE (*n* = 3). *p ≤ 0.05. (N–P) Western blot analyses and quantification of GBE1 in WT and *Gaa* ^−/−^ mice at 34 weeks in QF, HRT, and DP lysates respectively. Values from three independent mice were normalized to total protein and averaged. Data are shown as mean ± SE (*n* = 3). **p ≤ 0.01. (Q–T) Western blot analyses and quantification of UGP2 in WT and *Gaa* ^−/−^ mice at 34 weeks in QF, HRT, DP, and BR lysates respectively. Values from three independent mice were normalized to total protein and averaged. Data are shown as mean ± SE (*n* = 3). *p ≤ 0.05. KO, knockout. All target proteins were detected within the dynamic range (Figure S1).

GYG1 is encoded by the *Gyg1* gene. It participates in the initiation stage of glycogen synthesis acting as a primer to form glycogen (Figure 1A).^18^, ^19^ Western blot analysis indicated a > 2‐fold upregulation of GYG1 levels in QF and DP, and a slight (1.3‐fold) upregulation in BR of 34‐week‐old *Gaa* ^−/−^ mice, a time point at which Pompe disease‐induced muscle weakness is evident as measured with histochemical and functional analyses,^17^ whereas no significant difference was detected in HRT (Figure 1C–F).

Glycogen synthase (muscle isoform; GYS1) is the isoform of GYS1 expressed in skeletal muscle and other tissues, and takes part in the elongation stage of glycogen biosynthesis (Figure 1A).^20^, ^21^ No significant differences in total GYS1 expression were observed in QF, HRT, and DP between age‐matched WT and Gaa^−/−^ animals (Figure 1G–I). GYS1 was not detected in the BR (data not shown).

GLUT4 mediates glucose uptake into skeletal muscle (Figure 1A).^22^, ^23^ GLUT4 levels were found to be upregulated >2‐fold in QF of *Gaa* ^−/−^ mice compared with WT. There were no differences in GLUT4 levels between *Gaa* ^−/−^ and WT mice in HRT, DP, and BR tissues (Figure 1J–M).

GBE1 is encoded by the *Gbe1* gene. GBE1 enables the generation of branches during glycogen biosynthesis (Figure 1A).^24^ GBE1 was expressed at significantly increased levels in *Gaa* ^−/−^ QF (>4‐fold) and HRT (~2‐fold) compared with WT. Slightly increased GBE1 levels were observed in *Gaa* ^−/−^ DP although this was not statistically different (Figure 1N–P). GBE1 was not detected in brain tissue (data not shown). GBE1 enzyme activity was also increased in *Gaa* ^−/−^ QF and HRT (Figure S2). Interestingly, GBE1 activity was also significantly increased in *Gaa* ^−/−^ DP compared with WT (Figure S2), despite similar GBE1 protein levels between *Gaa* ^−/−^ and WT mice (Figure 1P).

UGP2 catalyzes the conversion of glucose‐1‐phosphate to UDP‐glucose (Figure 1A).^25^ UGP2 levels were slightly (~1.3 fold) but significantly upregulated in QF of *Gaa* ^−/−^ mice, whereas its levels were unchanged in *Gaa* ^−/−^ HRT, DP, and BR tissues (Figure 1Q–T).

Together, these results indicate that protein expression of enzymes involved in glycogen synthesis/glucose uptake including GYG1, GLUT4, GBE1, and UGP2 but not GYS1 in QF and GLUT4 in DP are upregulated in adult mice with Pompe disease‐induced loss of muscle mass and function.

### 2.2 Timing of disturbed cytoplasmic glycogen metabolism in *Gaa* ^−/−^ mice

We previously reported that loss of muscle mass in *Gaa* ^−/−^ mice (FVB/N) starts between 15 and 25 weeks of age.^17^ To determine the timing of metabolic changes relative to the development of functional changes, we next analyzed expression of the above enzymes in QF and HRT of mice before onset (at 10 weeks) and long after at advanced stage of disease (at 60 weeks).

**FIGURE 2:**
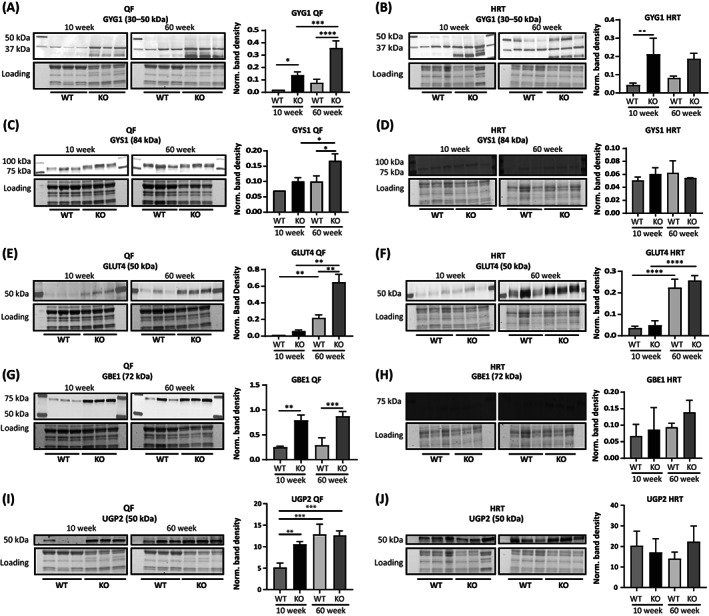
Timing of disturbed expression of glycogen metabolizing enzymes in *Gaa* ^−/−^ mice. (A,B) Western blot analyses and quantification of glycogenin (GYG1) in wild‐type (WT) and *Gaa* ^−/−^ mice at 10 and 60 weeks in quadriceps femoris (QF) and heart (HRT), respectively. To quantify GYG1 levels, all bands between 50 and 30 kDa were used. (C, D) Western blot analyses and quantification of glycogen synthase (GYS1) in WT and *Gaa* ^−/−^ mice at 10 and 60 weeks in QF and HRT, respectively. (E, F) Western blot analyses and quantification of glucose transporter 4 (GLUT4) in WT and *Gaa* ^−/−^ mice at 10 and 60 weeks in QF and HRT, respectively. (G, H) Western blot analyses and quantification of glycogen branching enzyme (GBE1) in WT and *Gaa* ^−/−^ mice at 10 and 60 weeks in QF and HRT, respectively. (I, J) Western blot analyses and quantification of UDP‐glucose pyrophosphorylase (UGP2) in WT and *Gaa* ^−/−^ mice at 10 and 60 weeks in QF and HRT, respectively. In all quantifications values from three independent mice were normalized to total protein and averaged. Data are shown as mean ± SE (*n* = 3). *p ≤ 0.05; **p ≤ 0.01; ***p ≤ 0.001; ****p ≤ 0.0001. KO, knockout. All target proteins were detected within the dynamic range (Figure S1).

Western blot analysis of GYG1 in QF revealed increased expression in *Gaa* ^−/−^ versus WT mice at both 10 weeks (7‐fold) and 60 weeks (~4.8‐fold) of age. In addition, GYG1 expression increased with age in both WT and *Gaa* ^−/−^ mice (Figure 2A). In the HRT, GYG1 expression was elevated in *Gaa* ^−/−^ mice at 10 weeks (>5‐fold) but not significantly at 60 weeks (Figure 2B). GYS1 levels were unchanged in *Gaa* ^−/−^ QF at 10 weeks, but increased 1.5‐fold in *Gaa* ^−/−^ QF at 60 weeks (Figure 2C). GYS1 levels were equal in HRT of both genotypes and at both ages (Figure 2D). GLUT4 expression was similarly low in QF muscles for both genotypes at 10 weeks, while at 60 weeks the levels were increased 3‐fold in *Gaa* ^−/−^ mice compared with WT mice (Figure 2E). In HRT, there was no difference between *Gaa* ^−/−^ and WT mice at any age (Figure 2F). GBE1 levels in *Gaa* ^−/−^ QF were upregulated ~4‐fold compared with WT at both 10 weeks and 60 weeks (Figure 2G). GBE1 differences in HRT were similar at both ages between both genotypes (Figure 2H). UGP2 levels were increased 2‐fold at 10 weeks, but not at 60 weeks, in QF of *Gaa* ^−/−^ mice compared with age‐matched WT counterparts (Figure 2I). In HRT, UGP2 levels were similar between WT and *Gaa* ^−/−^ mice at both ages (Figure 2J).

Taken together, in *Gaa* ^−/−^ QF, UGP2 was strongly upregulated at 10 weeks of age, before the loss of muscle function, while this upregulation attenuated with progression of pathology at 34 and 60 weeks; GYS1 and GLUT4 were upregulated at 34 and/or 60 weeks but not at 10 weeks; and GBE1 expression was upregulated at all ages analyzed. Results of this and previous studies were summarized in Table 1.

**TABLE 1:** Overview of in vivo studies evaluating glycogen metabolism in Pompe disease

	Species	Tissues	ERT	GYG1	GYS1	GLUT4	GBE1	UGP2	PYGM	HK1	pGS	G6P
Orth and Mundegar, 2003	Human	Not disclosed	No	—	—	↑ in Pompe	—	—	—	—	—	—
Douillard‐Guilloux et al.^10^	Mouse	Heart	No	—	Genetic inactivation of *Gys1* in a *Gaa*−/− mice rescued muscle function	—	—	—	—	—	—	—
		Gastrocnemius		—		—	—	—	—	—	—	—
		Tibialis anterior		—		↑ in *Gaa*−/− Normalized in *Gys1*−/−*Gaa*−/−	—	—	—	—	—	—
		Soleus		—		—	—	—	—	—	—	—
		EDL		—		—	—	—	—	—	—	—
Taylor et al.^11^	Mouse	Heart	Yes	—	↑ in *Gaa*−/− Normalized after ERT	—	—	—	—	↑ in *Gaa*−/− Normalized after ERT	↑ in *Gaa*−/−	↑ in *Gaa*−/− Normalized after ERT
		Triceps		↑ in *Gaa*−/− Normalized after ERT	↑ in *Gaa*−/− Normalized after ERT	—	—	—	Active form ↓ in *Gaa*−/−	↑ in *Gaa*−/− Normalized after ERT	↑ in *Gaa*−/−	↑ in *Gaa*−/− Normalized after ERT
		Quadriceps		—	↑ in *Gaa*−/−	—	—	—	—	—	↑ in *Gaa*−/−	—
		Liver		—	—	—	—	—	—	—	—	—
Baligand et al.^40^	Mouse	Gastrocnemius	rAAV‐GAA (intramuscular)	—	—	—	—	—	—	—	—	↑ in *Gaa*−/− Normalized after rAAV‐GAA
Meena et al.^15^	Mouse	Gastrocnemius	Yes	—	—	↑ in *Gaa*−/− Normalized after ERT	—	UDP‐glucose, (product of UGP2) ↑ in *Gaa*−/−	—	—	↑ in *Gaa*−/− Normalized after ERT	↑ in *Gaa*−/−
This study	Mouse	Quadriceps	No	↑ in *Gaa*−/−	↑ in *Gaa*−/−	↑ in *Gaa*−/−	↑ in *Gaa*−/−	↑ in *Gaa*−/−	—	—	—	—
		Diaphragm		↑ in *Gaa*−/−	= in *Gaa*−/− and WT	= in *Gaa*−/− and WT	= in *Gaa*−/− and WT	= in *Gaa*−/− and WT	—	—	—	—
		Heart		↑ in *Gaa*−/−	= in *Gaa*−/− and WT	= in *Gaa*−/− and WT	↑ in *Gaa*−/−	= in *Gaa*−/− and WT	—	—	—	—
		Brain		↑ in *Gaa*−/−	—	= in *Gaa*−/− and WT	—	= in *Gaa*−/− and WT	—	—	—	—
	Human	Quadriceps	Yes	Reduced after ERT^a^	Reduced after ERT^a^	—	↑ in Pompe Normalized after ERT^a^	= in Healthy and Pompe = in Baseline and ERT	—	—	—	—

Abbreviations: EDL, extensor digitorum longus; ERT, enzyme replacement therapy; G6P, glucose‐6‐phosphate, GAA, glucosidase; GBE1, glycogen branching enzyme; GLUT4, glucose transporter 4; GYS1, glycogen synthase; GYG1, glycogenin; HK1, hexokinase 1; pGS, phosphorylated glycogen synthase; PYGM, glycogen metabolizing enzymes like phosphorylase; UGP2, UDP‐glucose pyrophosphorylase.

_a_ Reduction in protein levels after paired analysis of each patient before and after ERT.

### 2.3 Cytoplasmic glycogen metabolism in human patients

To assess whether these findings observed in *Gaa* ^−/−^ mice could be extended to human patients with Pompe disease, we analyzed muscle biopsies from patients who were mildly affected at baseline and who showed a positive response to ERT within 2–3 years after start of treatment (Table S1). The reasons for this selection were (1) that it would most resemble the phenotype in the *Gaa* ^−/−^ mice, which develop a muscle phenotype at adulthood; (2) that biopsies with a very strong muscle pathology would have excessive loss of muscle tissue and possibly replacement with fat. Age‐matched healthy control biopsies were included in the analysis. To this end, biopsies taken from the QF of late‐onset pompe disease (LOPD) compound heterozygous c.‐32‐13 T > G (IVS1) patients (i.e., that carry the IVS1 variant on first allele and a disease‐associated *GAA* variant on the second allele) were selected (Table 2 and Table S1).

**TABLE 2:** Patient demographics

	Pompe	Control
Gender		
Male	20%	50%
Female	80%	50%
Age range in years (mean)	35–69 (50.8)	68–81 (72.7)
Follow‐up (months)		
20–30	60%	N/A
31–36	40%	N/A
Reduced muscle function (MRC score) at baseline		
75%–80%	40%	N/A
≥81%	60%	N/A

**FIGURE 3:**
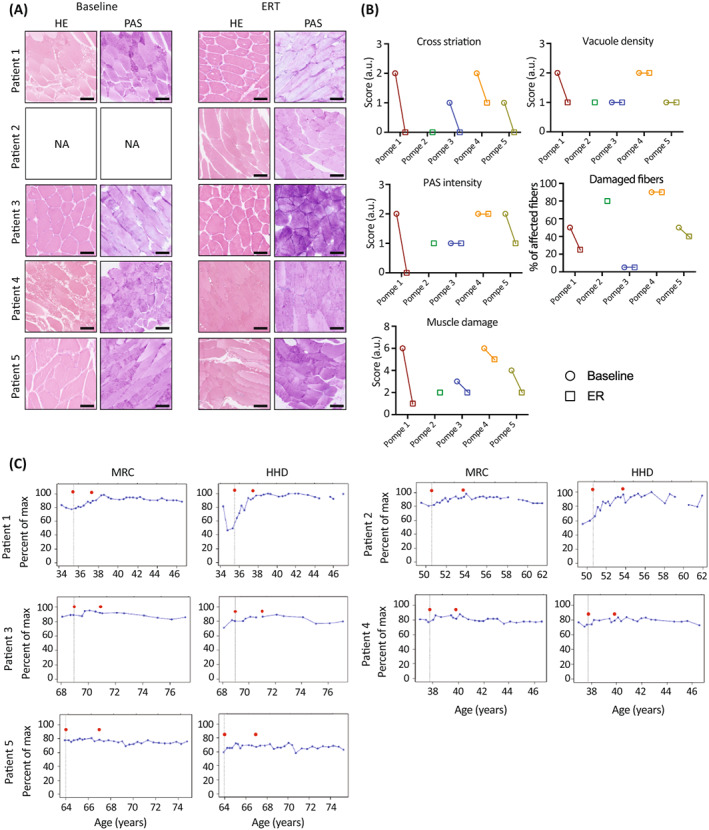
Histopathological and clinical evaluation of patient analyzed in this study. (A) Hematoxylin and eosin (HE) and periodic acid Schiff (PAS) staining of biopsies from quadriceps femoris in patients at baseline and after start of enzyme replacement therapy (ERT) treatment. Scale bars 100 μm. (B) Quantification of histological parameters by two independent researchers to assess muscle damage. HE and PAS stainings of patients at baseline and after start of ERT were used. *Baseline image of Patient 2 (Pompe 2) at baseline was not available. The figure depicts a consensus score for cross‐striation, vacuole density, PAS intensity. The percentage of damaged fibers was counted and averaged. The muscle damage score is the sum of cross‐striation, vacuole density, and PAS intensity. (C) Medical Research Council (MRC) and hand‐held dynamometry (HHD) scores of patients. Percentage of maximum force is represented in y‐axis. Age in years is represented in x‐axis. Black vertical lines with a red dot indicate the start of ERT. The second red dot indicates the time of the follow‐up biopsy.

Histopathological changes were analyzed using hematoxylin and eosin (HE)‐stained and periodic acid Schiff (PAS)‐stained skeletal muscle sections from patients at baseline as well as after start of ERT (Figure 3A). This revealed in patients at baseline the presence of vacuolar myopathy, alterations in fiber size and structure, disruption of cross striation, lysosomal enlargement, increased presence of round‐shaped glycogen‐filled lysosomes, and fat tissue replacement (Figure S3). These parameters were scored blindly by two independent researchers, according to quantitative and qualitative parameters further described in Table 3 and as described before.^17^, ^26^ On average all patients scored higher for disruption of cross‐striation at baseline (1.5 ± 0.6) compared with ERT treatment (0.2 ± 0.4), indicating a recovery of cross‐striation after start of ERT treatment (Figure 3B). The intensity of PAS staining was overall reduced after start of ERT comparted to that at baseline (1.8 ± 0.5 at baseline vs. 1 ± 0.7 at ERT) (Figure 3B). There were no changes in vacuolar density (1.5 ± 0.6 vs. 1.2 ± 0.4) or the percentage of affected fibers before and after start of ERT, except for Patient 1, who showed a reduction of damaged fibers after start of ERT treatment (Figure 3B). Muscle damage was calculated as the sum of all scores for each patient at baseline and after start of ERT, with higher scores indicating increased muscle damage. Overall, all patients analyzed scored lower for total muscle damage after treatment with ERT (4.8 ± 1.5 at baseline vs. 2.4 ± 1.5 at ERT) (Figure 3B). All patients showed improvement or stabilization of muscle function. MRC sum‐scores at baseline were on average 80.8% ± 4.7%, and this value was increased to 86.8% ± 6.5% upon treatment with ERT (Figure 3C). For hand‐held dynamometry (HHD) sum‐score baseline values were at 66.8% ± 10.5%, which were increased to 84.8 ± 13 after start of ERT (Figure 3C).

**FIGURE 4:**
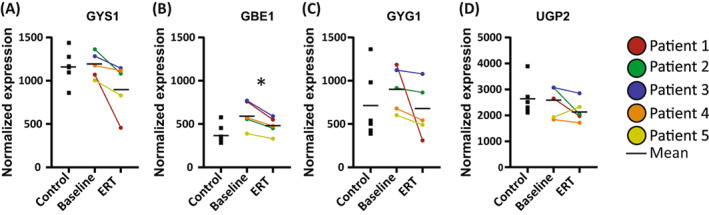
Quantitative mass spectrometry analysis of muscle biopsies from Pompe disease patients. (A–D) Normalized expression of glycogen synthase (muscle isoform; GYS1), glycogen branching enzyme (GBE1), glycogenin (GYG1), and UDP‐glucose pyrophosphorylase (UGP2) in Controls, Baseline, and enzyme replacement therapy (ERT) groups. Horizontal bars represent the mean for each group. *q = 0.15

Expression of metabolic proteins in patient and healthy control biopsies was assessed using quantitative mass spectrometry (MS) employing tandem mass tag labeling followed by liquid chromatography with tandem MS (LC‐MS/MS). Out of the five human orthologs of the mouse proteins studied here, four (GYS1, GBE1, GYG1, and UGP2) reached significant expression levels to be detected by mass spectrometry, leaving GLUT4 undetected. Large interindividual differences in individual expression levels of these proteins were found in both healthy controls and patients that ranged up to 3‐fold (Figure 4). The average expression levels of GYS1, GBE1, GYG1, and UGP2 were not statistically different between patients at baseline and healthy controls, nor between patients after ERT treatment and healthy controls. However, paired analysis of baseline versus ERT‐treated for each individual patient indicated that all patients downregulated GYS1 (fold change [FC] 0.78 ± 0.2), GBE1 (FC 0.8 ± 0.05), and GYG1 (FC 0.76 ± 0.29), and all but one patient downregulated UGP2 (FC 0.9 ± 0.21) in response to ERT. This suggests that expression of human GYS1, GBE1, GYG1, and UGP2, all of which are involved in cytoplasmic glycogen biosynthesis, is responsive to ERT in skeletal muscle of late‐onset Pompe disease patients that have a positive response to ERT, while it is not significantly different to healthy controls.

## 3 DISCUSSION

Lysosomes play important roles not only in degradation of complex molecules, but are also involved in many other processes such autophagy and cellular signaling. Loss of lysosomal function by accumulation of storage products may trigger a pathological cascade culminating in collapse of critical cellular functions.^27^ As one of such affected cellular processes, glycogen metabolism was found to be dysregulated in a mouse model of Pompe disease. We not only confirmed dysregulation of two glycogen metabolizing enzymes—GYG1 and GYS1—in limb muscle and heart of GAA‐deficient mice on a different genetic background as was used in previous studies, but identified two additional enzymes—GBE1 and UGP2—that were upregulated in GAA‐deficient tissue. Furthermore, our analyses demonstrated that glycogen metabolism—in particular GYG1—was also upregulated in DP and BR—additional tissues that are affected in Pompe disease. We found differential regulation of glycogen synthesis and glucose transport enzymes in muscle of mice before and after the onset of muscle wasting, which we previously have found to develop between 15 and 25 weeks in this mouse model.^17^ UGP2 was preferentially upregulated before the loss of muscle mass and function, GYG1, GLUT4, and GBE1 during progression of loss of muscle function, and GYS1 only when muscle loss was already advanced. To investigate if glycogen metabolism was also dysregulated in human Pompe disease, we analyzed muscle biopsies from five patients by quantitative mass spectrometry showing that the levels of GYS1, GBE1, GYG1, and UGP2—proteins that were upregulated in skeletal muscle of GAA‐deficient mice—decreased upon ERT treatment in all patients tested. Together our data show tissue‐specific and temporal changes in glycogen synthesis that may result in accumulation of glycogen also in the cytoplasm in addition to the excess present in the lysosome.

The current and previous studies that studied glycogen metabolism in Pompe disease used *Gaa* ^−/−^ mice, as mouse model of Pompe disease. The GAA‐knockout mouse model shares features with classic infantile Pompe disease—including accumulation of glycogen from birth and hypertrophic cardiomyopathy—but also—with respect to disease onset and progression—with LOPD.^16^, ^17^, ^28^ Previous studies used GAA‐deficient animals in a mixed BL6/129 background, while we analyzed tissues from *Gaa* ^−/−^ mice in an FVB/N background (see Table 1) for an overview of findings in this and previous studies). Raben and colleagues reported that in gastrocnemius muscle inactive phosphorylated GYS1 was increased more than total GYS1 in both young (<16 weeks) and old (>24 weeks) GAA‐deficient mice,^15^, ^29^, ^30^ suggesting that GYS1 activity would be reduced in *Gaa* ^*−/−*^ muscle. In contrast, Taylor *et al*.^11^ showed that total GYS1 increased more strongly than phosphorylated GYS1 in QF muscle from 16‐week GAA‐deficient mice. In our study, we also find a strong increase in total GYS1 in QF at 60 weeks animals (Figure 2), which may support an increase in GYS1 activity. In the study of Taylor *et al*. GYS1 levels were already upregulated in 16 week‐old GAA‐deficient mice in the Bl6/129 mixed background, while we studied GAA‐knockout animals on an FVB/N background and found GYS1 to be upregulated in QF muscle only at 60 weeks of age. These findings seem to indicate that the timing of changes in GYS1 levels may be dependent on genetic background.

Other members of glycogen metabolism were found consistently differentially expressed—across different muscles and host strains—in multiple studies. Elevated GLUT4 has also been found in tibialis anterior muscle of *Gaa* ^−/−^ (Bl6/129) mice by Douillard‐Guilloux *et al*.,^10^ and in human skeletal muscle biopsies from late‐onset patients by Orth and Mundegar.^10^, ^31^ Other evidence for disturbed cytoplasmic glycogen metabolism in *Gaa* ^−/−^ (Bl6/129) mice includes elevated activity of hexokinase and its activator glucose‐6‐phosphate in triceps and HRT, reduced levels of phosphorylase‐b (which degrades cytoplasmic glycogen at high adenosine monophosphate concentrations) in triceps, and increased levels of UDP‐glucose in gastrocnemius muscle.^11^, ^15^ In addition, ERT has been shown to revert increased expression of GYS1, hexokinase, and glucose 6‐phosphate levels in skeletal muscles of *Gaa* ^−/−^ mice.^11^ This study complements these previous observations by establishing that glycogen metabolism is dysregulated early during disease progression predominantly in limb skeletal muscle and to a lesser extent in HRT, DP, and BR. We cannot exclude sex differences in glycogen metabolism may exist, as mice were not sex‐matched in this study.

In this study, two new enzymes were identified that showed increased expression in skeletal muscle of *Gaa* ^−/−^ mice, GBE1 and UGP2. Together with GYS1 and GYG1, GBE1 regulates the last step of glycogen biosynthesis. GYG1 functions as a protein primer that initiates glycogen synthesis, GYS1 elongates the growing chains, GBE1 inserts side chains to branch the glycogen molecule^24^, and UGP2 catalyzes the critical conversion of glucose‐1‐phosphate to UDP‐glucose.^32^ All these enzymes were upregulated in skeletal muscle of Pompe disease mice, suggesting enhanced cytoplasmic glycogen build‐up, as reported previously.^13^ In addition to increasing glycogen levels, GBE1 may affect the structure of glycogen. It is known that reduced levels of GBE1, as observed in GSD IV (Andersen disease), alters the structure of glycogen, affecting its solubility and resulting in accumulation of insoluble glycogen.^33^, ^34^

The *Gaa* ^−/−^ (FVB/N) mouse model that was used for this study starts to accumulate biochemically detectable glycogen in tibialis anterior at least at 2 weeks of age.^16^, ^17^ Our previous studies show that the lysosomal muscle phenotype developed between 15 and 25 weeks in *Gaa* ^−/−^ mice. GYG1, GBE1, and UGP2 were already upregulated in QF muscle at 10 weeks of age in this study. As such, by analyzing tissues at different ages and stages of disease, these data indicate that a subset of enzymes involved in cytoplasmic glycogen biosynthesis is already upregulated before the onset of muscle wasting in skeletal muscle of *Gaa* ^−/−^ mice and may contribute to disease development. A limitation of this study is that the levels of cytoplasmic glycogen levels in tissues of *Gaa* ^−/−^ mice were not determined, and the potential pathological effect of increased cytoplasmic glycogen levels not tested. The experiments in this study indicate that cytoplasmic glycogen metabolism continues to increase during disease progression. This might contribute to the Pompe disease phenotype in two ways: (1) by increasing the availability of glycogen substrate for entry into the lysosomes. Indeed it has been demonstrated that glycogen synthesized in the cytoplasm can be taken up in the lysosomes by autophagy, in a process termed glycophagy.^35^ Further support for this mechanism has been obtained by Douillard‐Guilloux *et al*.^10^ who showed that inhibition of cytoplasmic glycogen synthesis by knockdown or knockout of GYS1 in a mouse model of Pompe disease resulted in reduced lysosomal glycogen accumulation. It should be noted that autophagy is also affected in Pompe disease,^3^, ^36^ so that it is unclear if cytoplasm‐derived glycogen continues to contribute to the increased levels of glycogen in the lysosome. In this study, we did not determine the role of disturbed autophagic activity on glycogen‐mediated pathology in Pompe disease; (2) by causing cytoplasmic glycogen accumulation. Cytoplasmic glycogen accumulation in human muscle biopsies from late‐onset patients with Pompe disease has been reported previously and may disrupt structural organization by displacing myofibrils.^13^ It has been suggested that cytoplasmic glycogen levels may also increase as result of lysosomal rupture,^37^ although it has not been determined to which extent this contributes to the total level of extralysosomal glycogen.

Our observations and those from the previous studies^10^, ^11^, ^15^, ^29^, ^30^ provide important insight into the pathophysiological mechanisms that contribute to disease progression in Pompe disease, but are restricted to mouse models. Our finding that the levels of GYG1, GYS1, GBE1, and UPG2 decreased in ERT‐treated samples of all patients (Figure 4; except for UGP2 in Patient 5) suggest that expression of these cytoplasmic glycogen synthesizing enzymes in human patients responded to ERT and that glycogen metabolism is also disrupted in human Pompe disease. We used a paired‐sample approach, in which samples before and after treatment are compared per patient, to minimize individual variation caused by differences in genetic backgrounds and increase the statistical power of the analysis. The large individual variation in protein expression was also observed in the healthy control samples. To assess physiological variability in enzyme expression, biopsies from healthy controls at different time points should be analyzed. Unfortunately, such samples were not available and hence the comparison of samples from (treated) patients with healthy controls had low statistical power. This large interindividual variation remains an obstacle for the study of human phenotypes using patient‐derived material. The response of glycogen metabolizing enzymes to ERT seems in line with observations in mice showing that ERT normalized dysregulated expression of GYS1, hexokinase, and glucose 6‐phosphate levels in skeletal muscles of *Gaa* ^−/−^ mice.^11^ In contrast, the levels of cytoplasmic glycogen seem not to be affected by ERT. A multicenter study in 16 late‐onset patients showed that—while lysosomal glycogen was cleared—cytoplasmic glycogen pools were still observed following ERT.^13^ This finding is consistent with GAA functioning at acidic pH of the lysosome^13^ and not at neutral pH in the cytoplasm and therefore it is unlikely that clear glycogen in the cytoplasm.

The patients that were selected for this study had a mild muscle phenotype, and also responded well to the first 2–3 years of ERT. This may suggest that mild changes in cytoplasmic glycogen metabolism in human patients are reversible. We speculate that the effect of ERT on cytoplasmic glycogen metabolism is indirect by decreasing lysosomal glycogen levels (and a subsequent normalization of cytoplasmic glucose levels), although we did not explore the presence of cytoplasmic glycogen in patients in this study, nor the mechanism involved. It is unlikely that recombinant human GAA used in ERT acts directly on cytoplasmic glycogen, as it is only active at acidic pH in lysosomes and is not discharged from endosomal compartments into the cytoplasm. This is possibly the reason why extralysosomal glycogen was not cleared after ERT treatment in a previous study.^13^

When muscle pathology has advanced beyond a certain stage, it might be difficult or impossible to reverse cytoplasmic build‐up.^13^ It is of note that mice nor patients in this study were subject to fasting conditions, which could have an impact on the results. This study was limited by the small sample size of patient biopsies and the mild phenotype of the patients that may have obscured more dramatic changes in glycogen metabolism. In mildly affected patients, the disease may be diagnosed at any age, excluding the possibility to study an age‐dependent effect in our study. Future studies should investigate a larger cohort of patients, including good and poor responders to ERT. Remaining questions include: how is cytoplasmic glycogen metabolism affected in classic infantile and severely affected late‐onset patients, do the levels of glycogen in the cytoplasm correlate with upregulation of glycogen biosynthesis and what is the effect of ERT treatment on glycogen metabolism in these patients?

## 4 METHODS

### 4.1 Ethical approval of study design

Animal experiments and procedures were approved by the local (Animal Experiments Committee [DEC]) and national (Central Committee for Animal Experiments [CCD]) animal experiment authorities in compliance with the European Community Council Directive guidelines (EU directive 86/609), regarding the protection of animals used for experimental purposes.

The Ethical Committee of the Erasmus MC University Medical Center approved the use of the biopsies for research purposes (MEC 2007–103). Written informed consent was obtained from all patients and control subjects or their legal guardians. Control biopsies were obtained from healthy subjects for which a progressive neuromuscular disorder was ruled out by medical history.

### 4.2 Collection of mouse tissue

QF, HRT, DP, and BR (whole brain excluding cerebellum) tissue were obtained from WT FVB/N (Envigo, The Netherlands) and *Gaa* ^−/−^ (in FVB/N background) animals at 10, 34, and 60 weeks.^16^, ^17^ Dissected tissues were flash‐frozen in liquid nitrogen‐cooled isopentane (Honeywell, Germany) and stored at −80°C until analysis. Animals of both sexes of both WT and Gaa^−/−^ animals were used. Mice were not fasted before tissue was collected.

### 4.3 Muscle biopsies and patient selection

Muscle biopsies were taken from *vastus lateralis* from five patients (age 35–69) and five controls (age 68–81) using a standard open surgery or needle biopsy procedure as described previously.^26^ Selected patients were diagnosed with Pompe disease with confirmed GAA enzyme deficiency, had symptom onset at adulthood and showed a positive response to ERT within 2–3 years after start of treatment as reflected by stabilization or improvement of histological and functional parameters.

All five patients carried the c.‐32‐13 T > G (IVS1) variant on one allele and a disease‐associated variant on the other. The average age at start of ERT in this group was 50 ± 12.5 years (average ± SD). Follow‐up biopsies were performed 2–3 years after initiating ERT treatment. The median time between baseline and follow‐up biopsy in this group was 2.6 ± 1.3 years. Patients did not fast before collection of muscle biopsy. Detailed description on histological condition and functional parameters of included patients can be found in Table S2.

### 4.4 Assessment of tissue section histology

HE and PAS stainings were performed on muscle sections that were processed into glycolmethacrylate as described in ref.^5^

### 4.5 Histological evaluation of muscle biopsies

Scoring of histological changes in muscle biopsies was previously described.^26^, ^38^ In short, all sections were evaluated by two researchers (Rodrigo Canibano‐Fraile and Robert M. Verdijk) who were blinded to the identity and clinical details of each patient. For all parameters, consensus scores were obtained and reported. Vacuole density was assessed using HE and PAS‐stained tissue sections. The changes in cross‐striation, PAS intensity, and tissue damage were assessed using PAS‐stained tissue sections. These levels were scored using a scale from 0 to 3 (Table 3). The percentage of damaged fibers was expressed as a percentage of the total number of fibers present in the section. The overall muscle damage score was expressed as the sum of cross‐striation, vacuole density, and PAS intensity.

**TABLE 3:** Muscle damage scoring system based on tissue pathology

Score	Cross striation	PAS intensity	Vacuolization
0	Normal	None	None
1	≥75% normal	Little in most or all	Little in all and/or significant in some
2	25%–75% normal	Significant in all and strong in some; significant or strong in most	Significant in all and/or many in some
3	≤25% normal	Very strong in all	Many in all

Abbreviation: PAS, periodic acid Schiff.

### 4.6 Protein and tissue analysis

Western Blot analysis was described before^39^ (detailed in Supplementary Methods S1). Samples were loaded within linear‐range of detection (Figure S1). Western Blot images were analyzed using Adobe Photoshop CS6 and FIJI (fiji.sc/Fiji). Histological sections were scanned on a Hamamatsu NanoZoomer 2.0 and Images were analyzed using NDP view software (v.2.5.19; Hamamatsu Photonics).

### 4.7 Enzyme activity assay for GBE1

Further detailed in Supplementary Methods S1.

### 4.8 Mass spectrometry

Details are thoroughly described in Supplementary Methods S1.

### 4.9 Statistics

Data are expressed as means ± SE. Normally distributed data for experiments with three or more independent groups were tested with one‐way analysis of variance followed by post hoc Tukey or Games–Howell correction for multiple tests. Non‐normally distributed data for experiments with three or more independent groups were tested with Kruskal–Wallis test. A *p*‐value of <0.05 was considered significant. Data were analyzed using IBM SPSS Statistics (version 26).

For the analysis of quantitative MS data, only proteins that registered an expression value for all the individuals were used. Paired *t*‐tests were conducted to analyze Baseline versus ERT samples. Unpaired *t*‐tests were conducted to analyze Healthy versus Baseline. Proteins were filtered using the gene ontology (GO) term “Glycogen metabolic process” (GO:0005977). Multiple testing correction was calculated using the Benjamini–Hochberg method. A *q*‐value of 0.2 was used as cutoff.

## AUTHOR CONTRIBUTIONS

Rodrigo Canibano‐Fraile designed the study, performed experiments, analyzed and interpreted the data, and participated in the preparation of the article. Carlos A. dos Santos, Marianne Hoogeveen‐Westerveld, and Jeroen A. A. Demmers performed experiments. Tim Snijders and Philip Lijnzaad assisted with the statistical analyses. Robert M. Verdijk analyzed and interpreted the data. Laurike Harlaar and Esther Brusse and interpreted the data. Nadine A. M. E. van der Beek, Pieter A. van Doorn, and Ans T. van der Ploeg interpreted the data. W. W. M. Pim Pijnappel and Gerben J. Schaaf designed the study, interpreted the data, and participated in the preparation of the article.

## FUNDING INFORMATION

The work was funded by a grant from the EU Joint Programme Neurodegenerative Disease research (JPND); and by a research grant from Sanofi Genzyme to the Center for Lysosomal and Metabolic Diseases at Erasmus MC.

## CONFLICT OF INTEREST

Ans T. van der Ploeg received research support for this paper from Sanofi Genzyme under an agreement with Erasmus MC University Medical Center. Data gathering and analysis were performed independently from the sponsor. All other authors declare no conflict of interest.

## INFORMED CONSENT

All procedures followed were in accordance with the ethical standards of the responsible committee on human experimentation (institutional and national) and with the Helsinki Declaration of 1975, as revised in 2000. Informed consent was obtained from all patients for being included in the study.

## ANIMAL RIGHTS

All institutional and national guidelines for the care and use of laboratory animals were followed.

## Supporting information


**FIGURE S1** Western blot analyses and quantification of glycogenin (GYG1), glycogen synthase (GYS1), glucose transporter 4 (GLUT4), glycogen branching enzyme (GBE1), and UDP‐glucose pyrophosphorylase (UGP2) to determine the range of detection of the antibodies. Protein lysates from quadriceps femoris of *Gaa* ^−/−^ mice at 40 weeks were used. In order to determine the optimal protein load for quantification of Western Blot data, the linearity of the antibodies was first assessed. In total, 5, 10, 20, and 40 μg of protein from mouse lysate were loaded in gels and blotted. The intensity of the signal was quantified and plotted. A total of 20 μg were taken as the optimal amount of protein.

**FIGURE S2** Enzyme activity assay of glycogen branching enzyme (GBE1) in wild‐type (WT) and *Gaa* ^−/−^ mice at 34 weeks in quadriceps femoris (QF), heart (HRT), and diaphragm (DP) lysates respectively. Values from three independent mice were normalized to total protein and averaged. Data are shown as mean ± SE (*n* = 3). *p ≤ 0.05; **p ≤ 0.01; ****p ≤ 0.0001.

**FIGURE S3** Analysis of hematoxylin and eosin and periodic acid Schiff (PAS) stainings allows evaluation of total muscle damage. Representative images are shown. * shows cross striation; # shows fiber myopathy, characterized by disorganized fiber architecture; ^ shows vacuolization; % shows areas of intense PAS positive staining and round‐shaped glycogen‐filled lysosomes.

**Table S1** Patient and muscle biopsy characteristics

**Table S1** Antibodies and dyes

**Appendix S1** Supporting Information

## Data Availability

A Supplementary File containing details of the methods for Mass Spectrometry measurement in addition to figures of other data obtained in this research, has been submitted. Other data presented in this study is available upon request from the corresponding author.

## References

[1] van der Ploeg AT , Reuser AJJ . Pompe's disease. Lancet. 2008;372(9646):1342‐1353. doi:10.1016/S0140-6736(08)61555-X 18929906

[2] Reuser AJJ , Hirschhorn R , Kroos MA . Pompe disease: glycogen storage disease type II, acid α‐glucosidase (acid maltase) deficiency. The Online Metabolic and Molecular Bases of Inherited Disease. McGraw Hill; 2018. doi:10.1036/ommbid.417

[3] Fukuda T , Ewan L , Bauer M , et al. Dysfunction of endocytic and autophagic pathways in a lysosomal storage disease. Ann Neurol. 2006;59(4):700‐708. doi:10.1002/ana.20807 16532490

[4] Schoser B , Stewart A , Kanters S , et al. Survival and long‐term outcomes in late‐onset Pompe disease following alglucosidase alfa treatment: a systematic review and meta‐analysis. J Neurol. 2017;264(4):621‐630. doi:10.1007/s00415-016-8219-8 27372449

[5] Van den Hout JMP , Kamphoven JHJ , Winkel LPF , et al. Long‐term intravenous treatment of Pompe disease with recombinant human alpha‐glucosidase from milk. Pediatrics. 2004;113(5):e448‐e457. 15121988 10.1542/peds.113.5.e448

[6] Kishnani PS , Nicolino M , Voit T , et al. Chinese hamster ovary cell‐derived recombinant human acid alpha‐glucosidase in infantile‐onset Pompe disease. J Pediatr. 2006;149(1):89‐97. doi:10.1016/j.jpeds.2006.02.035 16860134 PMC2692727

[7] Kishnani PS , Corzo D , Nicolino M , et al. Recombinant human acid [alpha]‐glucosidase: major clinical benefits in infantile‐onset Pompe disease. Neurology. 2007;68(2):99‐109. doi:10.1212/01.wnl.0000251268.41188.04 17151339

[8] Harlaar L , Hogrel J‐Y , Perniconi B , et al. Large variation in effects during 10 years of enzyme therapy in adults with Pompe disease. Neurology. 2019;93:e1756‐e1767. doi:10.1212/WNL.0000000000008441 31619483 PMC6946483

[9] de Vries JM , van der Beek NA , Hop WC , et al. Effect of enzyme therapy and prognostic factors in 69 adults with Pompe disease: an open‐label single‐center study. Orphanet J Rare Dis. 2012;7:73. doi:10.1186/1750-1172-7-73 23013746 PMC3519647

[10] Douillard‐Guilloux G , Raben N , Takikita S , et al. Restoration of muscle functionality by genetic suppression of glycogen synthesis in a murine model of Pompe disease. Hum Mol Genet. 2009;19:684‐696. doi:10.1093/hmg/ddp535 19959526 PMC6281383

[11] Taylor KM , Meyers E , Phipps M , et al. Dysregulation of multiple facets of glycogen metabolism in a murine model of Pompe disease. PLoS One. 2013;8(2):e56181. doi:10.1371/journal.pone.0056181 23457523 PMC3572993

[12] Douillard‐Guilloux G , Raben N , Takikita S , Batista L , Caillaud C , Richard E . Modulation of glycogen synthesis by RNA interference: towards a new therapeutic approach for glycogenosis type II. Hum Mol Genet. 2008;17(24):3876‐3886. doi:10.1093/hmg/ddn290 18782850

[13] van der Ploeg A , Carlier PG , Carlier RY , et al. Prospective exploratory muscle biopsy, imaging, and functional assessment in patients with late‐onset Pompe disease treated with alglucosidase alfa: the EMBASSY study. Mol Genet Metab. 2016;119:115‐123. doi:10.1016/j.ymgme.2016.05.013 27473031

[14] Raben N , Nagaraju K , Lee E , Plotz P . Modulation of disease severity in mice with targeted disruption of the acid α‐glucosidase gene. Neuromuscul Disord. 2000;10(4):283‐291. doi:10.1016/S0960-8966(99)00117-0 10838256

[15] Meena NK , Ralston E , Raben N , Puertollano R . Enzyme replacement therapy can reverse pathogenic Cascade in Pompe disease. Mol Ther Methods Clin Dev. 2020;18:199‐214. doi:10.1016/j.omtm.2020.05.026 32671132 PMC7334420

[16] Bijvoet AGA , van de Kamp EHM , Kroos MA , et al. Generalized glycogen storage and cardiomegaly in a knockout mouse model of Pompe disease. Hum Mol Genet. 1998;7(1):53‐62. doi:10.1093/hmg/7.1.53 9384603

[17] Schaaf GJ , van Gestel TJM , In't Groen SLM , et al. Satellite cells maintain regenerative capacity but fail to repair disease‐associated muscle damage in mice with Pompe disease. Acta Neuropathol Commun. 2018;6(1):119. doi:10.1186/s40478-018-0620-3 30404653 PMC6220463

[18] Roach PJ , Depaoli‐Roach AA , Hurley TD , Tagliabracci VS . Glycogen and its metabolism: some new developments and old themes. Biochem J. 2012;441:763‐787. doi:10.1042/BJ20111416 22248338 PMC4945249

[19] Hurley TD , Stout S , Miner E , Zhou J , Roach PJ . Requirements for catalysis in mammalian glycogenin. J Biol Chem. 2005;280(25):23892‐23899. doi:10.1074/jbc.M502344200 15849187 PMC1266300

[20] Manchester J , Skurat AV , Roach P , Hauschka SD , Lawrence JC . Increased glycogen accumulation in transgenic mice over expressing glycogen synthase in skeletal muscle. Proc Natl Acad Sci U S A. 1996;93(20):10707‐10711. doi:10.1073/pnas.93.20.10707 8855244 PMC38219

[21] Pederson BA , Chen H , Schroeder JM , Shou W , DePaoli‐Roach AA , Roach PJ . Abnormal cardiac development in the absence of heart glycogen. Mol Cell Biol. 2004;24(16):7179‐7187. doi:10.1128/mcb.24.16.7179-7187.2004 15282316 PMC479719

[22] Thorens B , Mueckler M . Glucose transporters in the 21st century. Am J Physiol Endocrinol Metab. 2010;298(2):141‐145. doi:10.1152/ajpendo.00712.2009 PMC2822486 20009031

[23] Cushman SW , Wardzala LJ . Potential mechanism of insulin action on glucose transport in the isolated rat adipose cell. Apparent translocation of intracellular transport systems to the plasma membrane. J Biol Chem. 1980;255(10):4758‐4762. 6989818

[24] Sean Froese D , Michaeli A , McCorvie TJ , et al. Structural basis of glycogen branching enzyme deficiency and pharmacologic rescue by rational peptide design. Hum Mol Genet. 2015;24(20):5667‐5676. doi:10.1093/hmg/ddv280 26199317 PMC4581599

[25] Perenthaler E , Nikoncuk A , Yousefi S , et al. Loss of UGP2 in brain leads to a severe epileptic encephalopathy, emphasizing that bi‐allelic isoform‐specific start‐loss mutations of essential genes can cause genetic diseases. Acta Neuropathol. 2020;139(3):415‐442. doi:10.1007/s00401-019-02109-6 31820119 PMC7035241

[26] Winkel LPF , Kamphoven JHJ , van den Hout HJMP , et al. Morphological changes in muscle tissue of patients with infantile Pompe's disease receiving enzyme replacement therapy. Muscle Nerve. 2003;27:743‐751. doi:10.1002/mus.10381 12766987

[27] Parenti G , Medina DL , Ballabio A . The rapidly evolving view of lysosomal storage diseases. EMBO Mol Med. 2021;13(2):e12836. doi:10.15252/emmm.202012836 33459519 PMC7863408

[28] Raben N , Nagaraju K , Lee E , et al. Targeted disruption of the acid alpha‐glucosidase gene in mice causes an illness with critical features of both infantile and adult human glycogen storage disease type II. J Biol Chem. 1998;273(30):19086‐19092. doi:10.1074/jbc.273.30.19086 9668092

[29] Raben N , Schreiner C , Baum R , et al. Suppression of autophagy permits successful enzyme replacement therapy in a lysosomal storage disorder‐‐murine Pompe disease. Autophagy. 2010;6(8):1078‐1089. doi:10.4161/auto.6.8.13378 20861693 PMC3039718

[30] Xu S , Lun Y , Frascella M , et al. Improved efficacy of a next‐generation ERT in murine Pompe disease. JCI Insight. 2019;4(5):10.1172/jci.insight.125358. 10.1172/jci.insight.125358 PMC6483515 30843882

[31] Orth M , Mundegar RR . Effect of acid maltase deficiency on the endosomal/lysosomal system and glucose transporter 4. Neuromuscul Disord. 2003;13(1):49‐54. doi:10.1016/S0960-8966(02)00186-4 12467732

[32] Turnquist RL , Turnquist MM , Bachmann RC , Hansen RG . Uridine diphosphate glucose pyrophosphorylase: differential heat inactivation and further characterization of human liver enzyme. BBA Enzymol. 1974;364(1):59‐67. doi:10.1016/0005-2744(74)90132-6 4433565

[33] Thon VJ , Khalil M , Cannon JF . Isolation of human glycogen branching enzyme cDNAs by screening complementation in yeast. J Biol Chem. 1993;268(10):7509‐7513. doi:10.1016/s0021-9258(18)53204-x 8463281

[34] Bruno C , Cassandrini D , Assereto S , Akman HO , Minetti C , di Mauro S . Neuromuscular forms of glycogen branching enzyme deficiency. Acta Myol. 2007;26(1):75‐78. 17915577 PMC2949312

[35] Zirin J , Nieuwenhuis J , Perrimon N . Role of autophagy in glycogen breakdown and its relevance to chloroquine myopathy. PLoS Biol. 2013;11(11):e1001708. doi:10.1371/journal.pbio.1001708 24265594 PMC3825659

[36] Nascimbeni AC , Fanin M , Masiero E , Angelini C , Sandri M . The role of autophagy in the pathogenesis of glycogen storage disease type II (GSDII). Cell Death Differ. 2012;19:1698‐1708. doi:10.1038/cdd.2012.52 22595755 PMC3438501

[37] Thurberg BL , Lynch Maloney C , Vaccaro C , et al. Characterization of pre‐ and post‐treatment pathology after enzyme replacement therapy for Pompe disease. Lab Invest. 2006;86(12):1208‐1220. doi:10.1038/labinvest.3700484 17075580

[38] Schaaf GJ , van Gestel TJ , Brusse E , et al. Lack of robust satellite cell activation and muscle regeneration during the progression of Pompe disease. Acta Neuropathol Commun. 2015;3(65):1‐11. doi:10.1186/s40478-015-0243-x 26510925 PMC4625612

[39] Demirdas S , van Slegtenhorst MA , Verdijk RM , et al. Delayed diagnosis of Danon disease in patients presenting with isolated cardiomyopathy. Circ Genomic Precis Med. 2019;12(3):e002395. doi:10.1161/CIRCGEN.118.002395 30919683

[40] Baligand C , Todd AG , Lee‐McMullen B , et al. 13C/31P MRS metabolic biomarkers of disease progression and response to AAV delivery of hGAA in a mouse model of Pompe disease. Mol Ther Methods Clin Dev. 2017;7:42‐49. doi:10.1016/j.omtm.2017.09.002 29018835 PMC5626920

