# Supplementary figures and images for: Lysosomal glycogen accumulation in Pompe disease results in disturbed cytoplasmic glycogen metabolism
Source: J Inherit Metab Dis. 2022 Oct 17;46(1):101–15. doi: 10.1002/jimd.12560 (PMC10092494; doi:10.1002/jimd.12560)

Canibano-Fraile et al. - Supplementary Figure 1

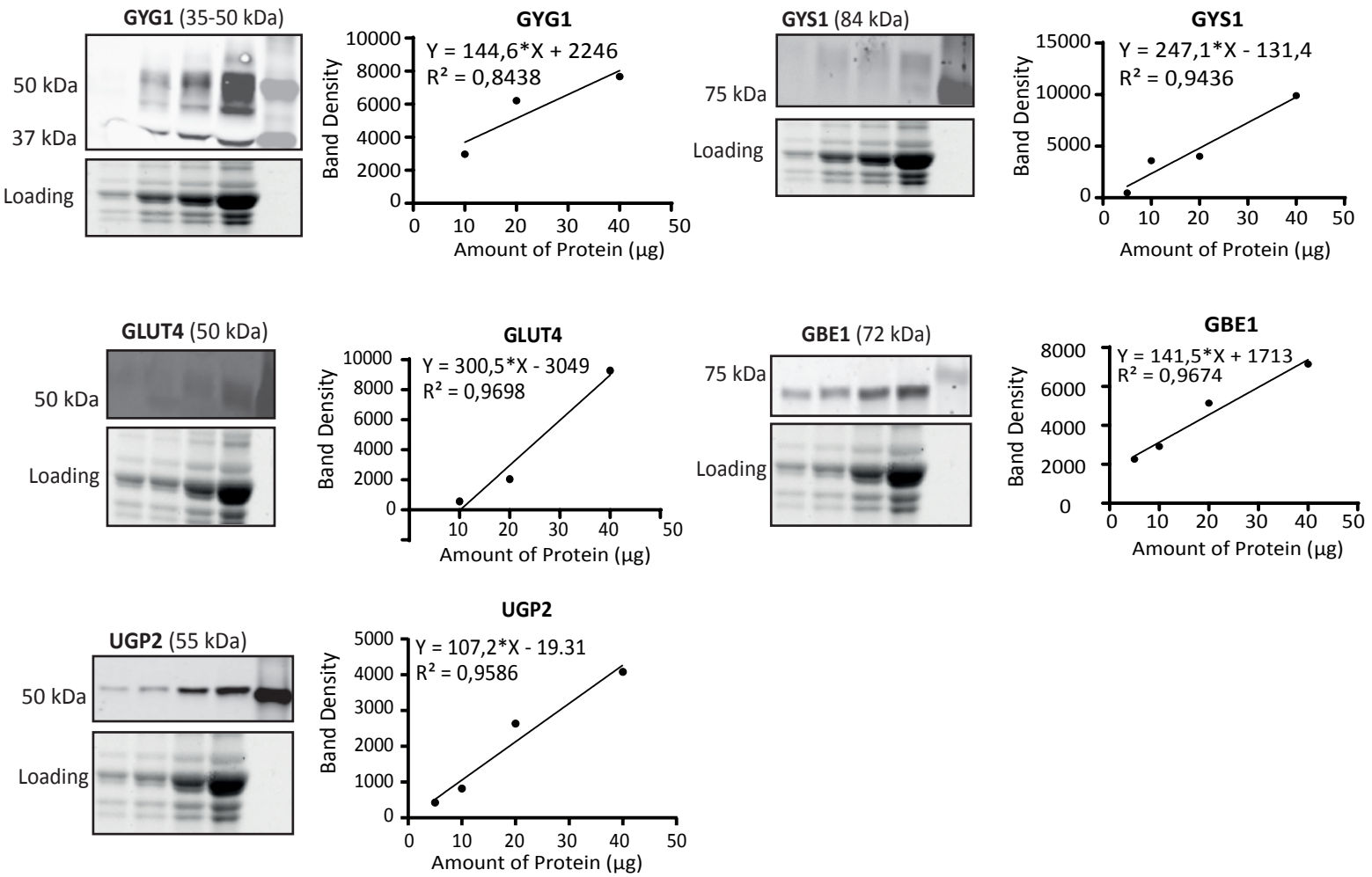

Supplement: Supplementary file 1 — FIGURE S1 Western blot analyses and quantification of glycogenin (GYG1), glycogen synthase (GYS1), glucose transporter 4 (GLUT4), glycogen branching enzyme (GBE1), and UDP‐glucose pyrophosphorylase (UGP2) to determine the range of detection of the antibodies. Protein lysates from quadriceps femoris of Gaa −/− mice at 40 weeks were used. In order to determine the optimal protein load for quantification of Western Blot data, the linearity of the antibodies was first assessed. In total, 5, 10, 20, and 40 μg of protein from mouse lysate were loaded in gels and blotted. The intensity of the signal was quantified and plotted. A total of 20 μg were taken as the optimal amount of protein. [file JIMD-46-101-s003.pdf]

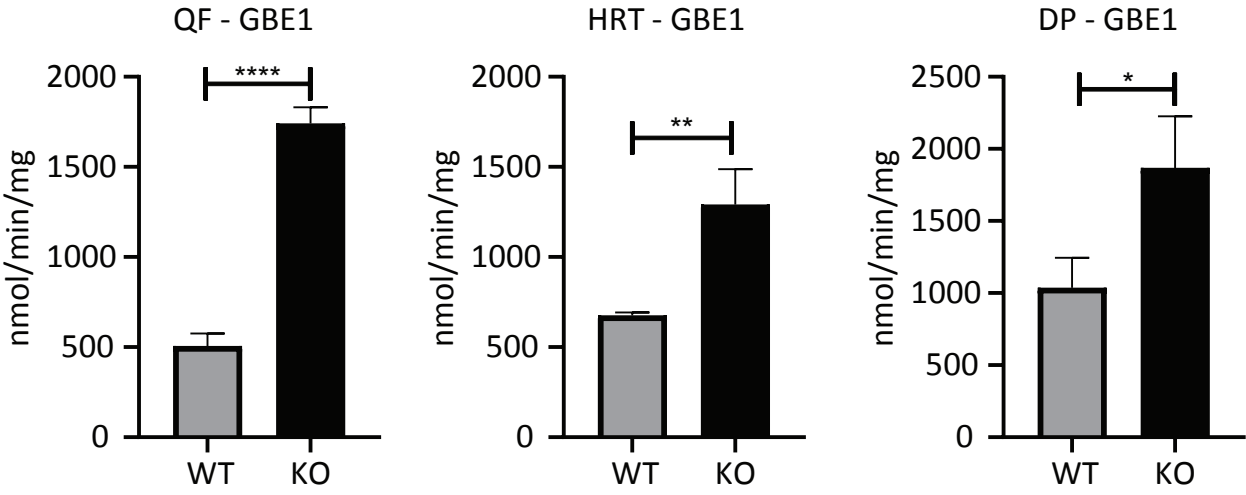

Supplement: Supplementary file 2 — FIGURE S2 Enzyme activity assay of glycogen branching enzyme (GBE1) in wild‐type (WT) and Gaa −/− mice at 34 weeks in quadriceps femoris (QF), heart (HRT), and diaphragm (DP) lysates respectively. Values from three independent mice were normalized to total protein and averaged. Data are shown as mean ± SE (n = 3). *p ≤ 0.05; **p ≤ 0.01; ****p ≤ 0.0001. [file JIMD-46-101-s004.pdf]

Canibano-Fraile et al. - Supplementary Figure 3

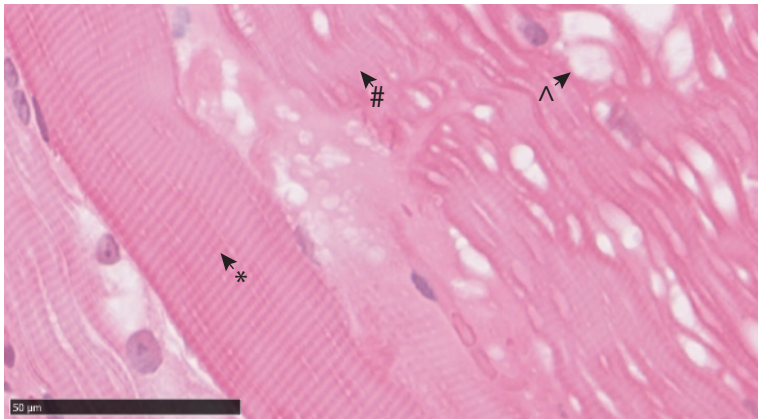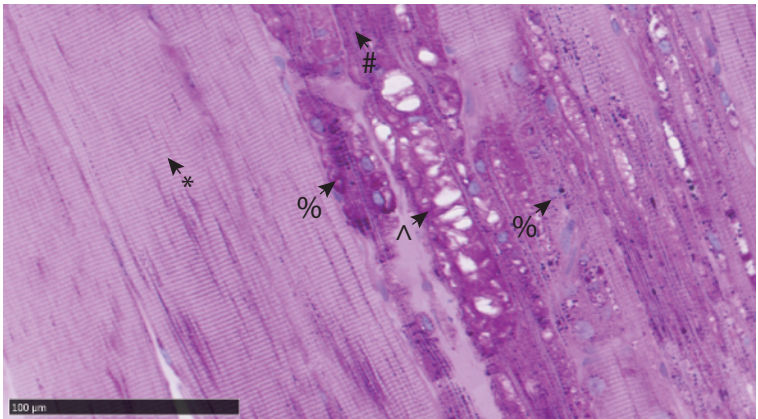

Supplement: Supplementary file 3 — FIGURE S3 Analysis of hematoxylin and eosin and periodic acid Schiff (PAS) stainings allows evaluation of total muscle damage. Representative images are shown. * shows cross striation; # shows fiber myopathy, characterized by disorganized fiber architecture; ^ shows vacuolization; % shows areas of intense PAS positive staining and round‐shaped glycogen‐filled lysosomes. [file JIMD-46-101-s006.pdf]
