# Supplementary material for: Lysosomal glycogen accumulation in Pompe disease results in disturbed cytoplasmic glycogen metabolism
Source: J Inherit Metab Dis. 2022 Oct 17;46(1):101–15. doi: 10.1002/jimd.12560 (PMC10092494; doi:10.1002/jimd.12560)
Supplement: Supplementary file 4 — Table S1 Patient and muscle biopsy characteristics [file JIMD-46-101-s005.docx]

| **Muscle biopsy donor ID** | **Gender** | **Age at diagnosis** | **Time in ERT at ERT biopsy (months)** | **MRC at baseline (% of max)** | **Increase in MRC after ERT (%)** | **HHD at baseline (% of max)** | **Increase in HHD after ERT (%)** |
| --- | --- | --- | --- | --- | --- | --- | --- |
| Patient 1 | Female | 35 | 25 | 80 | 10 | 50 | 40 |
| Patient 2 | Female | 50 | 35 | 82 | 12 | 64 | 33 |
| Patient 3 | Male | 69 | 23 | 90 | 4 | 80 | 7 |
| Patient 4 | Female | 37 | 25 | 79 | 5 | 78 | 3 |
| Patient 5 | Female | 63 | 36 | 78 | 0 | 60 | 8 |
| Control 1 | Male | 68 | - | - | - | - | - |
| Control 2 | Female | 77 | - | - | - | - | - |
| Control 3 | Male | 68 | - | - | - | - | - |
| Control 4 | Male | 69 | - | - | - | - | - |
| Control 5 | Female | 73 | - | - | - | - | - |
| Control 6 | Female | 81 | - | - | - | - | - |

**Supplementary Table 1:** Patient and muscle biopsy characteristics
