# Supplementary material for: Lysosomal glycogen accumulation in Pompe disease results in disturbed cytoplasmic glycogen metabolism
Source: J Inherit Metab Dis. 2022 Oct 17;46(1):101–15. doi: 10.1002/jimd.12560 (PMC10092494; doi:10.1002/jimd.12560)
Supplement: Supplementary file 5 — Table S1 Antibodies and dyes [file JIMD-46-101-s002.docx]

**Table S2. Antibodies and dyes**

| **Antibody** | **Dilution** | **Info** |
| --- | --- | --- |
| Mouse anti-GYG1 | 1:500 | Novus Bio (Cat#: H00002992-M07).  RRID: AB_539428 |
| Rabbit anti-GYS1* | 1:1000 | Cell Signaling (Cat#: 3886S)  RRID: AB_2116392 |
| Rabbit anti-GLUT4 | 1:1000 | Abcam (Cat#: ab654)  RRID: AB_305554 |
| Rabbit anti-GBE1 | 1:1000 | Atlas Antibodies (Cat#: HPA038074)  RRID: AB_10672403 |
| Mouse anti-UGP2 | 1:250 | Santa Cruz (Cat#: sc-514174)  RRID: N/A |
| Goat anti-Rabbit IRDye 800CW | 1:5000 | Li-Cor (Cat#: 925-32211)  RRID: AB_2651127 |
| Goat anti-Mouse IRDye 800CW | 1:5000 | Li-Cor (Cat#: 925-32210)  RRID: AB_2687825 |
| Goat anti-Rabbit IRDye 680RD | 1:5000 | Li-Cor (Cat#: 925-68071)  RRID: AB_2721181 |
| Goat anti-Mouse IRDye 680RD | 1:5000 | Li-Cor (Cat#: 925-68070)  RRID: AB_2651128 |

*: This antibody recognizes total unphosphorylated GYS1, which corresponds to total GYS1.
