# Supplementary material for: Lysosomal glycogen accumulation in Pompe disease results in disturbed cytoplasmic glycogen metabolism
Source: J Inherit Metab Dis. 2022 Oct 17;46(1):101–15. doi: 10.1002/jimd.12560 (PMC10092494; doi:10.1002/jimd.12560)
Supplement: Supplementary file 6 — Appendix S1 Supporting Information [file JIMD-46-101-s001.docx]

**Supplementary Legends**

**Supplementary Figure 1.** Western Blot analyses and quantification of GYG1, GS, GLUT4, GBE1, and UGP2 to determine the range of detection of the antibodies. Protein lysates from QF of *Gaa*^-/-^ mice at 40 weeks were used. In order to determine the optimal protein load for quantification of Western Blot data, the linearity of the antibodies was first assessed. 5, 10, 20, and 40 µg of protein from mouse lysate were loaded in gels and blotted. The intensity of the signal was quantified and plotted. 20 µg were taken as the optimal amount of protein.

S**upplementary Figure 2.** Enzyme activity assay of GBE1 in WT and *Gaa*^-/-^ mice at 34 weeks in QF, HRT, and DP lysates respectively. Values from three independent mice were normalized to total protein and averaged. Data are shown as mean ± SE. n=3. *p≤0.05; **p≤0.01; ****p≤0.0001.

**Supplementary Figure 3.** Analysis of HE and PAS stainings allows evaluation of total muscle damage. Representative images are shown. * shows cross striation; # shows fiber myopathy, characterized by disorganized fiber architecture; ^ shows vacuolization; % shows areas of intense PAS positive staining and round-shaped glycogen-filled lysosomes.

**Supplementary Methods**

**Histological features and functional parameters of included patients.**

Patients showed disease-associated histological changes in muscle biopsies and reduced scores on clinical assessments regarding muscle function and strength at baseline. Histological features in patients’ muscle biopsies – as evaluated (see below) by two researchers, including an experienced neuropathologist – included increased vacuolization, increased PAS-positive staining, cross-striation, and a reduced number of healthy fibers. Skeletal muscle strength was measured using the Medical Research Council (MRC) grading scale and by hand-held dynamometry (HHD, Cytec dynamometer, Groningen, the Netherlands) [40]. The following muscle groups were tested for either method: neck extensors, neck flexors, shoulder abductors, elbow flexors, elbow extensors, hip flexors, hip abductors, knee flexors, and knee extensors. In addition, the MRC grade was determined for shoulder adductors, shoulder exorotators and endorotators, hip extensors, and hip adductors. This was expressed as the percentage of the maximum possible score for MRC sum scores, and as the percentage of the median strength of healthy males and females for HHD sum scores. Selected patients scored less than 90% on manual muscle testing function using MRC grading and less than 80% in HHD score at baseline.

**Western Blot Analysis**

Tissue was mechanically homogenized and lysed in RIPA buffer supplemented with a phosphatase inhibitor cocktail (10 mM NaF, 60 mM β-glycerolphosphate, 2 mM Na-orthovanadate) and cOmplete^TM^ protease inhibitor cocktail (Sigma-Aldrich). Equal amounts – mostly 20 μg - of protein were resolved on 4-15% Criterion^TM^ TGX^TM^ precast gels (BioRad Laboratories) and transferred to nitrocellulose membranes. Nitrocellulose membranes were blocked in 5% milk diluted in TBS Tween 0.1% (TBST), 5% bovine serum albumin (BSA) and incubated in primary antibody, washed extensively in TBST, and incubated in IRDye labelled-secondary antibody (Li-cor). Membranes were read on an Odyssey reader (Li-Cor).

**Enzyme activity assay for GBE1**

10% (w/v) muscle homogenates in 50 mM aquous NaF were prepared using an ultrasonic homogenizer (VCX 130, Sonics, Newtown CT, USA). Samples were centrifuged 10 minutes at 10000 rpm at 4°C. Pellets were discarded. Total protein concentration was determined using Pierce^TM^ BCA Protein Assay Kit according to manufacturer’s instructions (Thermo Scientific, Rockford IL, USA). Lysates were diluted in 50 mM NaF to a concentration of 2 mg/ml. 10 µl of 2x lysate were diluted in 100 µl of reaction mix (25 µg/ml glycogen; 18.5 mM glycine buffer (0.2 M glycylglycine/NaOH, pH 6.4); 5 mM AMP; 50 mM 2-mercapto ethanol, 50 mM glucose-1-phosphate (G1P); 0.3 mg/ml phosphorylase B). Samples were incubated at 30°C for 90 minutes. Reactions were stopped by incubation on ice. Free inorganic phosphate was measured in a spectrophotometer (Cary 300, Varian/Agilent Technologies) using MoFe-reagent (144 mM FeSO_4_.7H_2_O).

**Mass Spectrometry**

Human skeletal muscle biopsies were cut and lysed in 1 ml 50 mM Tris/HCl pH 8.2, 0.5 % sodium deoxycholate (SDC) and MS-SAFE™ protease and phosphatase inhibitor using a Bioruptor ultasonicator (Diagenode). Protein concentrations were measured using the BCA assay (Thermo Scientific). Proteins were reduced with 5 mM DTT and cysteine residues were alkylated with 10 mM iodoacetamide. Protein was extracted by acetone precipitation at -20 °C overnight. Samples were centrifuged at 8,000 g for 10 min at 4 °C. The acetone was removed and the pellet allowed to dry. The protein pellet was dissolved in 1 ml 50 mM Tris/HCl pH 8.2, 0.5 % SDC and proteins were digested with LysC (1:200 enzyme:protein ratio) for 4 h at 37 °C. Next, trypsin was added (1:100 enzyme:protein ratio) and the digestion proceeded overnight at 30 °C. Digests were acidified with 50 μl 10 % formic acid (FA) and centrifuged at 8,000 g for 10 min at 4 °C to remove the precipitated SDC. The supernatant was transferred to a new centrifuge tube. The digests were purified with C18 solid phase extraction (Sep-Pak, Waters), lyophilized and stored at -20 °C.

Proteolytic peptides were labeled with TMT 10-plex labeling reagents (Thermo Scientific) allowing for peptide quantitation. Peptides were mixed at the 10-plex level and further fractionated by HILIC chromatography. Fractions were collected and analyzed by nanoflow LC-MS/MS. nLC-MS/MS was performed on EASY-nLC 1200 coupled to an Orbitrap Lumos Tribid mass spectrometer (Thermo) operating in positive mode and equipped with a nanospray source. Peptides were separated on a ReproSil C18 reversed phase column (Dr Maisch GmbH; column dimensions 15 cm × 50 µm, packed in-house) using a linear gradient from 0 to 80% B (A = 0.1 % formic acid; B = 80% (v/v) acetonitrile, 0.1 % formic acid) in 70 min and at a constant flow rate of 200 nl/min using a splitter. The column eluent was directly sprayed into the ESI source of the mass spectrometer. Mass spectra were acquired in continuum mode; fragmentation of the peptides was performed in data-dependent mode using the multinotch SPS MS3 reporter ion-based quantification method.
